# Development of clinical research networks in rural America: Our experience from the Accelerating COVID-19 Therapeutic Interventions and Vaccines-1 trialau

**DOI:** 10.1017/cts.2025.10208

**Published:** 2025-12-17

**Authors:** Eyal Kedar, Dima Dandachi, Alexis Bryant, Kevin J. Anstrom, William Powderly

**Affiliations:** 1 Division of Rheumatology, Department of Clinical and Rural Health Research, St. Lawrence Health, Canton, NY, USA; 2 Division of Infectious Diseases, University of Missouri, Columbia, MO, USA; 3 Department of Biostatistics, University of North Carolina, Chapel Hill, NC, USA; 4 Division of Infectious Diseases, Washington University, St. Louis, MO, USA

**Keywords:** Clinical research networks, rural healthcare, master protocols, clinical research Training, healthcare disparities

## Abstract

Rural America remains deeply under-represented in clinical trials. St Lawrence Health (SLH) was the sole rural site and one of the top recruiters in the Accelerating COVID-19 Therapeutic Interventions and Vaccines (ACTIV)-1 trial, which was a large international trial that studied the efficacy of three immune modulators in hospitalized patients with COVID-19. In this article, we analyze the structural and clinical factors that enabled SLH’s success in the context of previously described barriers to research participation in rural areas. We conclude with lessons learned from the SLH experience and offer a broader replicable model for developing clinical research capacity in rural areas. SLH’s success in ACTIV-1 can be attributed to early and sustained support from the ACTIV-1 network, a small and integrated inpatient COVID-19 treatment team, regular and consistent communication between this team and the clinical research team at SLH, and SLH’s ongoing support and development of its clinical research department. SLH was, in turn, able to overcome several known barriers to implementation of clinical trials at community sites, including lack of provider time and a lack of trained research and clinical staff, and its experience in ACTIV-1 offers a replicable model for developing clinical research capacity in rural communities.

## Background

In 2025, American rural communities continue to be deeply under-represented in clinical trials [1]. In 2022, residents living in rural areas made up 13.8% of the United States (U.S.) population [2], yet the percentage of rural Americans participating in clinical trials is substantially lower [3]. As an example, a study evaluating geographic differences in cancer treatment trial availability found that 86% of non-metropolitan counties had no trials registered on ClinicalTrials.gov in 2022 compared to 44% of metropolitan counties [4]. While the advent of tools such as electronic data capture and decentralized trials hold promise for increasing research accessibility, the rural urban research gap remains wide.

Many factors make the provision of healthcare in general and implementation of clinical trials particularly challenging in rural areas [5,6]. These include: (1) Individual and community barriers such as lower health literacy, social stigma, mistrust of healthcare systems, and lack of rural community engagement in research; (2) Organizational barriers such as limited investment in research infrastructure and marked shortages of medical specialists and trained researchers working in rural locations, and limited collaboration between rural health systems and academic and government research networks; and (3) Systematic barriers such as lower patient volumes, higher poverty rates, lower insurance rates, underemployment, technical difficulties with limited transportation, inadequate internet access, and decreased cellphone connectivity [6–12].

Without systematic and comprehensive strategies to address barriers to research participation as well as coordinated and sustained efforts between stakeholders, rural America is likely to continue to fall behind not only in clinical research participation [10] but in its health outcomes overall [11].

The Accelerating COVID-19 Therapeutic Interventions and Vaccines (ACTIV)-1 trial was an international, multi-center master protocol that evaluated the efficacy of three immune modulators, abatacept, cenicriviroc, and infliximab, in hospitalized patients with COVID-19. It was coordinated by the National Institutes of Health (NIH) as part of a public–private partnership that included the Biomedical Advanced Research and Development Authority (BARDA), the Centers for Disease Control, and various other representatives from the U.S. government, academia, and the pharmaceutical industry [13,14]. As an inpatient trial conducted during one of the deadliest phases (the pre-Omicron era) of the COVID-19 pandemic, ACTIV-1 presented a number of challenges to its clinical sites. These included a relative lack of clinician time [15] and a need for often daily care coordination between inpatient teams and nursing, pharmacy and research staff.

St. Lawrence Health (SLH) consists of a flagship hospital, Canton Potsdam Hospital (CPH), and two critical access hospitals in the rural North County region of New York. It is based in St Lawrence County, the largest and most medically remote county in New York and also one of the poorest counties in the state [16]. SLH has a catchment area that extends to several surrounding rural counties and which includes over 150,000 patients. CPH is three hours by car to the closest tertiary care medical center but heavy snowfall often makes travel to tertiary care centers challenging during the winter months.. Of the 48 U.S. sites that participated in ACTIV-1, SLH was the only site with its largest hospital in a Federal Office of Rural Health Policy designated rural county [17]. Despite this and SLH’s relatively small size (under 150 hospital beds across its three hospitals during the ACTIV-1 trial), SLH was among the top enrolling sites in the ACTIV-1 trial, not just within the U.S., but across all participating international sites.

SLH utilized a hub and spoke model during the ACTIV-1 trial in which all patients seen in any of its emergency rooms with severe COVID-19 who were clinically stable for transfer were transferred and admitted to CPH and subsequently managed by the SLH inpatient COVID-19 treatment team [8]. As a result, there were often over 25 to 30 hospitalized COVID-19 patients on the daily census at CPH (which then had a total bed capacity of only 94 beds) during the most severe waves of the pandemic in the pre-Omicron era. The SLH inpatient COVID-19 treatment team faced numerous barriers to enrolling patients in ACTIV-1 which have previously been described in other surveys of health care workers [18] and which included a lack of clinical staff with training in research and a small (just six team members) clinical research team. The result was that SLH clinicians and research staffers working on the ACTIV-1 trial frequently worked for weeks or months on end without a break. And yet, in spite of these challenges, SLH was one of ACTIV-1’s top international recruiters. This success can be attributed to regular communication with and support from ACTIV-1 leaders, daily SLH inpatient COVID-19 treatment meetings that included the clinical research and inpatient pharmacy teams at SLH, a strong sense of mission among SLH staffers involved in the trial, careful discussion of the risks and benefits of participating in the trial between the SLH ACTIV-1 inpatient treatment team and hospitalized COVID-19 patients, and sustained internal support from the SLH administration for its clinical research department. Although there was a high risk of burnout among treatment and research team members, SLH was nonetheless able to maintain a zero attrition rate for both of these teams throughout the trial. Its experience in ACTIV-1 offers a replicable model for developing clinical research capacity in rural communities.

## The SLH model during the ACTIV-1 trial

Several factors contributed to SLH’s success in ACTIV-1. SLH’s spoke and hub model likely reduced systematic barriers to research participation in rural areas including the barriers of potentially lower patient volumes (all COVID-19 patients eligible for transfer among SLH’s three hospitals were transferred to CPH) and difficulties with transportation. SLH’s inpatient COVID-19 team was small and consisted of several generalist and specialist physicians (initially a hospitalist, an infectious disease physician and a rheumatologist with experience in treating interstitial lung disease, and eventually a pulmonologist), several APP’s (hospitalist, infectious disease) and an inpatient pharmacist [8]. The team met daily, including on weekends, and had frequent discussions about how best to describe the trial to patients and their families in a way that was respectful, non-coercive and clear. The team had minimal personnel turnover (only the hospitalist rotated weekly; all other team members remained constant). This consistency and continuity in care facilitated trial coordination and patient recruitment and led to strong knowledge of the ACTIV-1 master protocol among the members of the COVID-19 treatment team, which in turn may have promoted patient trust and willingness to participate in ACTIV-1. There was a strong relationship between the clinical and research team members, who often spoke once or several times per day about the trial. Although there was a clear risk of burnout for team members working without reinforcements, team members commonly spoke about a sense of mission in their work and so it is possible that the team’s selection process (investigators and sub-investigators had to complete several hours of research training including good clinical practice training) naturally selected for clinicians who would be more mission-driven and less prone to burnout.

Another factor in SLH’s success was the development of its clinical research program. Though initiating a new research program would likely have been impossible during a pandemic, SLH had already had over five years of experience as a clinical trials site before the ACTIV-1 trial began. This momentum, and the interest and willingness of ACTIV-1’s leaders to include a small rural site, helped SLH overcome the common organizational barriers of low investment in research infrastructure and limited collaboration with larger research networks and, in turn, almost certainly contributed to its success in ACTIV-1. As SLH’s support of its clinical research program was publicized in a combination of local and national media outlets [19,20]. this may have also helped reduce additional individual and community research barriers such as mistrust of healthcare systems, a lack of community awareness of clinical trials, perceived lack of time and money on the part of some patients for participation in research, and social stigma.

In the future, formation of additional partnerships similar to that formed with the ACTIV-1 network as well as incorporation of validated frameworks for evaluating site performance (such as participation in the Reach, Effectiveness, Adoption, Implementation, Maintenance, or RE-AIM, network [21]) will likely play important roles in the development of SLH’s clinical research department and may, in turn, provide additional lessons in how to best develop and protect rural clinical research departments.

### Developing clinical research capacity in rural areas

An ideal model for increasing participation of rural sites (particularly sites that are not affiliated with academic centers and which often care for some of the most medically underserved populations) in clinical trials must go beyond building research infrastructure. It should also focus on developing a workforce of clinicians and clinician researchers who are well connected to larger industry and government research networks [22]. This model is what enabled SLH, despite its small size and location in a poor rural county, to succeed and make a substantial contribution in the ACTIV-1 trial. Replicating this success will require a targeted and ideally coordinated effort.

One such effort is in the direction of subspecialty internal medicine training in academic medical centers. This training is increasingly focused on training micro-specialists with a deep and narrow area of clinical and/or research interest. Although such a model (i.e. the one disease super-specialist model) is often effective in producing effective researcher-clinicians, it is also paradoxically more likely to deepen the shortage of internal medicine (e.g., cardiology, gastroenterology, pulmonology, rheumatology, etc.) subspecialty care in rural areas. This shortage disproportionately impacts medically disadvantaged populations, including American Indians who reside in rural areas and who were highly overrepresented in the SLH arm at 8% of participants (SLH, in this case, serves Franklin County, which includes part of the Akwesasne Mohawk Reservation within its boundaries and which has the highest percentage of Native Americans per capita of any county in New York State at 8.8% of the population [23]) of the ACTIV-1 trial (Table 1). Another challenge is that clinical training programs based in rural areas and/or with rural tracks are almost exclusively focused on developing primary care physicians (i.e. family medicine, internal medicine, OB-GYN, pediatrics) and often offer either minimal or no clinical research training [24]. Among primary care residencies, the current effort is particularly focused on training family medicine physicians [25]. As of January 2023, the University of Washington Alaska Internal Medicine Rural Residency Program became the nation’s first accredited rural track program in Internal Medicine [26]. While this focus addressed some healthcare needs, there remains a severe shortage of medical subspecialists in the rural healthcare workforce. In 2022, as one example, the number of cardiologists per 100,000 in urban areas was nearly seven times higher than rural areas, and the number of gastroenterologists was more than eight times greater [27]. The National Rural Health Association, furthermore, reports that rural communities in the U.S. have fewer than one ninth the number of medical specialists per capita than non-rural communities [28].

**Table 1.** Demographic and Baseline Characteristics of patients enrolled in ACTIV-1 at St. Lawrence Health (SLH) and overall ACTIV-1 population

Demographics and baseline characteristics		St. Lawrence Health (N = 50)	Total Randomized (N=1,971)
Age, mean (SD), years		63.3 (11.9)	54.8 (14.6)
Sex, no. (%)	Female	20 (40.0)	753 (38.2)
	Male	30 (60.0)	1218 (61.8)
Race, no. (%)	American Indian or Alaska Native	4 (8.0)	19 (1.0)
	Asian	0	56 (2.8)
	Black or African American	0	274 (13.9)
	Native Hawaiian or other Pacific Islander	0	2 (0.1)
	White	46 (92.0)	1229 (62.4)
	Multiple	0	7 (0.4)
	Other	0	279 (14.2)
	Unknown	0	105 (5.3)
Disease Severity at Baseline, no. (%)	Severe	18 (36.0)	858 (43.5)
	Moderate	32 (64.0)	1113 (56.5)
Clinical Status (8-point Ordinal Scale) at Baseline, no. (%)	2: Hospitalized, on invasive mechanical vent. or ECMO	1 (2.0)	191 (9.7)
	3: Hospitalized, on non-invasive vent. or high flow oxygen	17 (34.0)	667 (33.8)
	4. Hospitalized, requiring supplemental oxygen	31 (62.0)	1026 (52.1)
	5: Hospitalized, not requiring supplemental oxygen - requiring in-patient care	1 (2.0)	87 (4.4)
Time from Symptom Onset, mean (SD)		−9.2 (4.3)	−11 (4.6)
BMI, mean (SD)		34.7 (8.5)	32.6 (8.2)
Obesity (BMI >= 30 kg/m^2^), no. (%)		33 (66.0)	1108 (57.3)
History of Hypertension, no. (%)		27 (54.0)	812 (41.2)
History of Diabetes Mellitus, no. (%)		15 (30.0)	542 (27.5)
History of Cancer, no. (%)		5 (10.0)	126 (6.4)
History of Coronary Artery Disease, no. (%)		12 (24)	128 (6.5)
History of Heart Failure, no. (%)		5 (10)	63 (3.2)

Other physician barriers to clinical trial participation include lack of awareness of clinical trials and patient eligibility, difficulties in initiating a trial discussion, lack of time and concerns of financial burdens [18,29–32] and inadequate funding opportunities for sites interested in establishing investigator-initial rural health research programs. Although programs such as the NIH’s Institutional Development Award (IDeA) program and its component program CoBRE (Centers for Biomedical Research Excellence) have been developed to help reduce geographic disparities in federal research funding [33,34], these programs still have limited reach (IDeA only funds residents of 23 states).

Addressing the rural research gap will require training a balanced rural healthcare workforce that includes not only family medicine clinicians, but also general internists, medical subspecialists, and advanced practice providers with clinical and in some cases research expertise who are committed to living and working in rural communities.

To build this capacity and to continue supporting them throughout their careers, a multifaceted approach is needed. This approach could include:

1. Positioning rural clinicians, particularly those who practice and conduct research, in leadership positions within research institutions and funding bodies. This will ensure that policy, funding priorities, and programmatic development are focused on community needs in rural areas and are informed by frontline experience.
2. Implementing early-stage education and training; Investing in pathway programs in high schools and colleges to expose rural students to careers in medicine and research to foster their interest; developing summer research internships for students from rural backgrounds and medical schools with dedicated rural health missions and departments; Increasing rural tracks in primary care and surgery residencies, and developing rural-specific clinical and research tracks in internal medicine subspecialty fellowships [35–37].
3. Enabling flexible educational models: For rural providers already in practice, research certificate programs for practicing clinicians and other healthcare professionals would allow them to gain research competency without requiring full-time academic enrollment.
4. Supporting networks and mentorship programs to establish academic–rural partnerships that promote research collaboration.

### Expanding the rural research workforce beyond traditional investigator roles

Not all healthcare providers wish to become researchers and independent investigators. However, as with the example of SLH, a team-based approach that fosters multiple levels of engagement is needed. Healthcare providers not interested in conducting independent research can still:

1. Support patient identification and recruitment.
2. Provide input on protocol feasibility in real-world clinical workflows.
3. Contribute clinical data to existing studies and be recognized as collaborators.

### Research infrastructure support

Clinical research in rural areas can further be facilitated by:

1. Developing centralized IRB, regulatory, and data coordination support for rural investigators.
2. Encouraging and simplifying the requirements for rural investigators to have adjunct academic appointments.
3. Creating new funding streams to provide protected research time and long-term support for rural clinician researchers [38].
4. Developing new high impact medical journals that prioritize solution-focused rural health research.
5. Supporting joint projects that address prioritized health issues by rural communities and rural clinicians with feasible implementation in resource-limited settings.

Barriers to clinical participation specific to rural communities should be at the forefront of these discussions. The development of clinical research capacity in rural areas will be best achieved within tailored and location-specific partnerships that, as with our experience in ACTIV-1, began in rural communities and include effective partners from academia, government and industry (such as, in our experience, investigators from a variety of academic medical centers, the NIH and pharmaceutical companies).

Ultimately, however, any such efforts will need to be implemented with an understanding of the specific needs of rural communities and of the resource limitations of rural sites [39–42]. With this, rural research will need to be driven by those who experience these challenges firsthand, with academic and government partners providing support rather than centralized control.

## Conclusion

The development of rural clinical research infrastructure should begin in rural communities with a combination of tailored and context-specific strategies that ensure both scientific rigor and community relevance. Although every clinical research department is unique, a key lesson from SLH’s experience in the ACTIV-1 trial is that rural research capacity can be successfully developed and expanded when local clinical teams are empowered and effectively partnered with national networks. In the future, such networks should include the integration of rural clinicians with experienced rural health and clinical researchers in academic centers [43]. Other ideas for increasing clinical research capacity in rural areas include providing government (state and/or federal) subsidies for clinical research time, increasing participation of rural sites in national programs such as the IDeA State Consortium for a Clinical Research Resource Center and the U.S. Department of Health and Human Services’ ARPH-A Advancing Clinical Trial Readiness programs, and developing structures that ensure that clinical research developed through such initiatives is being done with the support rather than the leadership of peers from non-rural communities.

In the experience of SLH in the ACTIV-1 trial, the integration of small clinical and research teams resulted in the daily consistent communication that is essential to effective recruitment in a clinical trial. Furthermore, although the SLH patients in the ACTIV-1 trial had significantly higher rates of heart failure, cancer, coronary artery disease, hypertension and obesity relative to the overall ACTIV-1 population (Table 1), and although lower vaccination rates, lower health literacy and higher mortality were common in many rural areas in the pandemic, the combination of strong communication within SLH and the support of the larger ACTIV-1 network enabled SLH to serve as an effective partner in this trial.

The best rural health solutions are those that respond to community needs. Recruitment success is best achieved when there is trust between clinical staff and patients, which is particularly important in hospitals serving historically marginalized populations. This was the context in which SLH’s inpatient COVID-19 treatment team and clinical trials program were both developed. And yet, it was only with the collaboration of national partners that SLH was able to join the ACTIV-1 trial and provide an invaluable clinical trial option to its patients. Just as SLH required a combination of local and national collaboration and support to participate in ACTIV-1, so does American rural healthcare now require an integrated local and national effort to succeed [40,41]. It is perhaps easiest to imagine clinical and research solutions in rural communities as being embedded with one another. Stated differently, we will need to train rural subspecialists, rural primary care physicians, and rural clinician researchers in tandem. The best training programs will be those that work to develop networks of primary care and subspecialty physicians as well as clinician-researchers who can become and remain funded to solve core rural health problems.

These programs may or may not be best housed within academic centers. It is possible, given the current barriers for trainees with an interest in becoming rural clinicians and/or researchers, that certificate and mentorship programs make more sense than dedicated rural clinical and research tracks in fellowship programs. It is also possible that solutions from abroad (such as the Australian model of having dedicated rural health departments in certain medical schools, decentralized training for medical students interested in rural practice, rural training pipelines that include specialist physicians, and a proportionally much larger investment in rural healthcare relative to the U.S. [44–46] or the U.S. (such as expanding medical schools focused on rural health to include their own subspecialty and clinical research training programs) will be part of the answer. None of these solutions, however, seems practical without the effort of the U.S. government. As one of the rare remaining political issues with broad bipartisan support, perhaps the next step begins with an expansion of the federal Rural Partners Network to include a planned partnership with funding agencies, medical schools, independent training programs, and key medical journals to form a realistic and viable plan for developing integrated clinical care and clinical research networks that work together to not only define but also to solve rural health problems.

Advancing rural health research must begin by developing clinical research capacity in rural communities. Future solutions will need to include a combination of novel approaches to training rural clinician researchers and integrating them into research delivery networks. The experience of SLH in ACTIV-1 offers an example which other rural health systems can use to help surmount common research barriers, develop their own clinical trial programs, and ultimately form successful partnerships in larger research networks.

## References

[1] Mudaranthakam DP , Gajewski B , Krebill H , et al. Barriers to clinical trial participation: Comparative study between rural and urban participants. JMIR Cancer. 2022;8:e33240. doi: 10.2196/33240. 35451964 PMC9073606

[2] Davis JC , Cromartie J , Farrigan T , Genetin B , Sanders A , Winikoff JB. Rural america at a glance 2023, 10.32747/2023.8134362.ers, edition (Report No. EIB-261). U.S. Department of Agriculture, Economic Research Service. 2023.

[3] Blake KD , Moss JL , Gaysynsky A , Srinivasan S , Croyle RT. Making the case for investment in rural cancer control: an analysis of rural cancer incidence, mortality, and funding trends. Cancer Epidemiol Biomarkers Prev. 2017;26:992–997. 28600296 10.1158/1055-9965.EPI-17-0092 PMC5500425

[4] Kirkwood MK , Schenkel C , Hinshaw DC , et al. State of geographic access to cancer treatment trials in the United States: are studies located where patients live? JCO Oncol Pract.2025;21:427–437. doi: 10.1200/OP.24.00261. 39356976 PMC11925346

[5] US Food and Drug Administration. Diversity of Clinical Trials Participation. (https://www.fda.gov/patients/clinical-trials-what-patients-need-know/diversity-clinical-trial-participation) Accessed March 27, 2024.

[6] Dandachi D , Reece R , Wang EW , Nelson T , Rojas-Moreno C , Shoemaker DM. Treating COVID-19 in rural America. J Rural Health. 2021;37(205–206. doi: 10.1111/jrh.12457. 32362035 PMC7267414

[7] Tanner A , Kim SH , Friedman DB , Foster C , Bergeron CD. Barriers to medical research participation as perceived by clinical trial investigators: communicating with rural and African American communities. J Health Commun. 2015;20:88–96. doi: 10.1080/10810730.2014.908985. 25204763

[8] Kedar E , Scott R , Soule DM , et al. COVID-19 in a rural health system in New York - case series and an approach to management. Rural Remote Health. 2021;21:6464. doi: 10.22605/RRH6464. 34253026

[9] Lovelett C , Medeiros M , Jaremczuk D , et al. Developing patient-centered outcomes research infrastructure in a rural community through patient and stakeholder engagement and education during the COVID-19 pandemic. J Clin Transl Sci. 2022;6:e143. doi: 10.1017/cts.2022.486. 36590347 PMC9794966

[10] Kim SH , Tanner A , Friedman DB , Foster C , Bergeron CD. Barriers to clinical trial participation: a comparison of rural and urban communities in South Carolina. J Community Health Jun. 2014;39:562–571. doi: 10.1007/s10900-013-9798-2. 24310703

[11] Rural Health Information Hub. Social Determinants of Health for Rural People (https://www.ruralhealthinfo.org/topics/social-determinants-of-health) Accessed July 04, 2024.

[12] Watson SE , Smith P , Snowden J , et al. Facilitators and barriers to pediatric clinical trial recruitment and retention in rural and community settings: a scoping review of the literature. Clin Transl Sci. 2022;15:838–853. doi: 10.1111/cts.13220. 35037409 PMC9010274

[13] O’Halloran JA , Ko ER , Anstrom KJ , et al. Abatacept, cenicriviroc, or infliximab for treatment of adults hospitalized with COVID-19 pneumonia: a randomized clinical trial. JAMA. 2023;330:328–339. doi: 10.1001/jama.2023.11043. 37428480 PMC10334296

[14] Collins FS , Stoffels P. Accelerating COVID-19 therapeutic interventions and vaccines (ACTIV): an unprecedented partnership for unprecedented times. JAMA. 2020;323:2455–2457. doi: 10.1001/jama.2020.8920. 32421150

[15] https://www.mgma.com/mgma-stats/nearly-half-of-america-s-doctors-are-busier-than-ever-as-covid-19-recovery-continues, Accessed 7/23/25.

[16] https://northcountrynow.com/stories/one-in-five-children-in-st-lawrence-county-below-poverty-line-report-says,2831, Accessed 10/17/25.

[17] HRSA. Rural Hospital Programs. (https://www.hrsa.gov/rural-health/grants/rural-hospitals) Accessed 02/09/2024.

[18] Smith TR , McCulloh R , Bui MT , et al. Perceived barriers to pediatric clinical trials implementation: a survey of health care staff. Kans J Med,. 2022;15:189–193. doi: 10.17161/kjm.vol15.15885. 35646254 PMC9126861

[19] https://www.northcountrypublicradio.org/news/story/42913/20210114/how-st-lawrence-health-system-is-researching-treatments-to-help-covid-19-patients-locally, Accessed 10/17/25.

[20] https://dailyyonder.com/rural-health-system-part-of-operation-warp-speed-trials-for-new-treatments/2021/02/15/, Accessed 10/17/25.

[21] Gaglio B , Rabin B , Smith ML , Porter GC , Ory MG , Estabrooks PA. REAIM planning and evaluation framework: adapting to new science and practice with a 20-year review. Front Public Health. 2013;7:64. doi: 10.3389/fpubh.2019.00064. PMC6450067 30984733

[22] Wohl DA , Adam SJ , Gibbs KW , et al. Engaging communities in therapeutics clinical research during pandemics: experiences and lessons from the ACTIV COVID-19 therapeutics research initiative. J Clin Transl Sci. 2024;8:e156. doi: 10.1017/cts.2024.561. 39540112 PMC11557280

[23] Franklin County Demographics. (https://www.censusdots.com/race/franklin-county-ny-demographics) Accessed 07/06/2025.

[24] Rural Residency Programs and Rural Rotation Sites., https://ruralgme.org/rural-programs, Accessed 07/06/2025.

[25] The RTT Collaborative. Accredited Rural Physician Residency Programs., https://rttcollaborative.net/rural-programs/residency-map/, Accessed 05/27/2025.

[26] https://akmedres.uw.edu/apply, Accessed 10/17/25.

[27] Orgera K , Senn S , Grover A. Rethinking Rural Health. Washington, DC: AAMC; 2023, 10.15766/rai_xmxk6320

[28] National Rural Health Association., https://www.ruralhealth.us/about-us/about-rural-health-care, Accessed 07/06/2025.

[29] Manne S , Kashy D , Albrecht T , et al. Attitudinal barriers to participation in oncology clinical trials: factor analysis and correlates of barriers. Eur J Cancer Care (Engl). 2015;24:28–38. doi: 10.1111/ecc.12180. Epub 2014-01-28. 24467411 PMC4417937

[30] Somkin CP , Ackerson L , Husson G , et al. Effect of medical oncologists attitudes on accrual to clinical trials in a community setting. J Oncol Pract. 2013;9:e275–e283. doi: 10.1200/JOP.2013.001120. Epub 2013-10-22. 24151327 PMC5706122

[31] Becevic M, Sheets LR, Wallach, E, etal, Telehealth and Telemedicine in Missouri. Mo Med, (2020). May-Jun 2020;117(3):228–234. PMC7302013 32636555

[32] Hollander JE , Carr BG. . Virtually perfect? Telemedicine for covid-19. N Engl J Med. 2020;382:1679–1681. doi: 10.1056/NEJMp2003539. 32160451

[33] Schaller MD. Efficacy of centers of biomedical research excellence (CoBRE) grants to build research capacity in underrepresented states. FASEB J. 2024;38:e23560. doi: 10.1096/fj.202301610R. 38498349

[34] Caulder E , Zhang J , Nagarkatti M , Nagarkatti P. Geographic inequality in funding by national institutes of health negatively impacts almost one-half of the states in the United States. Front Public Health. 2024;12:1452494. doi: 10.3389/fpubh.2024.1452494. 39386949 PMC11461335

[35] University of Minnesota. CentraCare St. Cloud family medicine residency 10/09/2023, https://med.umn.edu/familymedicine/education-training/residency/st-cloud.

[36] University of Washington. WWAMI rural health research center 10/09/2023, https://www.washington.edu/research/research-centers/washington-wyoming-alaska-montana-idaho-wwami-rural-health-research-center/.

[37] School of Medicine. University of missouri. Rural Health Research Center. 08/02/2023, (https://medicine.missouri.edu/centers-institutes-labs/rural-health-research-center) Accessed December 2, 2025.

[38] https://www.gih.org/philanthropy-work/featured/biden-harris-administration-announces-75-million-investment-in-rural-health-care/, Accessed 7/23/25.

[39] Kwon SC , Tandon SD , Islam N , Riley L , Trinh-Shevrin C. Applying a community-based participatory research framework to patient and family engagement in the development of patient-centered outcomes research and practice. Transl Behav Med. 2018;8:683–691. doi: 10.1093/tbm/ibx026. 30202926 PMC6128966

[40] Mullins CD , Abdulhalim AM , Lavallee DC. Continuous patient engagement in comparative effectiveness research. JAMA. 2012;307;1587–1588. doi: 10.1001/jama.2012.442. 22511684

[41] Frank L , Basch E , Selby JV , Institute P-COR. The PCORI perspective on patient-centered outcomes research. JAMA. 2014;312:1513–1514. doi: 10.1001/jama.2014.11100. 25167382

[42] Wheat JR , Leeper JD , Murphy S , Brandon JE , Jackson JR. Educating physicians for rural america: validating successes and identifying remaining challenges with the rural medical scholars program. J Rural Health. 2018;4 Suppl 31:s65–s74. doi: 10.1111/jrh.12236. 28318061

[43] Kedar E. Commentary: a specific recommendation for improving healthcare – the rural generalist. The Daily Yonder, https://dailyyonder.com/commentary-a-specific-recommendation-for-improving-healthcare-the-rural-generalist/2023/06/28/

[44] Australian Government, Department of Health and Agent Care., https://www.health.gov.au/sites/default/files/2024-06/the-bonded-medical-program-student-information-booklet.pdf, Accessed 7/6/2025.

[45] Woolley T , Sen Gupta T , Murray R. James Cook University’s decentralised medical training model: an important part of the rural workforce pipeline in northern Australia. Rural Remote Health. 2016;16:3611. doi: 10.22605/RRH3611. 26992830

[46] Australian Government Department of Health, Disability and Ageing., https://www.health.gov.au/our-work/specialist-training-program, Accessed 7/6/2025.

