# Development of clinical research networks in rural America: Our experience from the Accelerating COVID-19 Therapeutic Interventions and Vaccines-1 trial – CORRIGENDUM

**DOI:** 10.1017/cts.2025.10233

**Published:** 2026-01-07

**Authors:** Eyal Kedar, Dima Dandachi, Alexis Bryant, Kevin J. Anstrom, William Powderly

In the original publication there was an error at the end of the title. The title was originally published as Development of clinical research networks in rural America: Our experience from the Accelerating COVID-19 Therapeutic Interventions and Vaccines-1 trialau. The ‘au’ has now been removed. This has been updated in the original article.
